# The Effects of Suction and Pin/Lock Suspension Systems on Transtibial Amputees’ Gait Performance

**DOI:** 10.1371/journal.pone.0094520

**Published:** 2014-05-14

**Authors:** Hossein Gholizadeh, Noor Azuan Abu Osman, Arezoo Eshraghi, Sadeeq Ali

**Affiliations:** Department of Biomedical Engineering, Faculty of Engineering, University of Malaya, Kuala Lumpur, Malaysia; Delft University of Technology (TUDelft), Netherlands

## Abstract

**Background:**

The suction sockets that are commonly prescribed for transtibial amputees are believed to provide a better suspension than the pin/lock systems. Nevertheless, their effect on amputees’ gait performance has not yet been fully investigated. The main intention of this study was to understand the potential effects of the Seal-in (suction) and the Dermo (pin/lock) suspension systems on amputees’ gait performance.

**Methodology/Principal Findings:**

Ten unilateral transtibial amputees participated in this prospective study, and two prostheses were fabricated for each of them. A three-dimensional motion analysis system was used to evaluate the temporal-spatial, kinematics and kinetics variables during normal walking. We also asked the participants to complete some part of Prosthesis Evaluation Questionnaire (PEQ) regarding their satisfaction and problems with both systems. The results revealed that there was more symmetry in temporal-spatial parameters between the prosthetic and sound limbs using the suction system. However, the difference between two systems was not significant (*p*<0.05). Evaluation of kinetic data and the subjects’ feedback showed that the participants had more confidence using the suction socket and the sockets were more fit for walking. Nevertheless, the participants had more complaints with this system due to the difficulty in donning and doffing.

**Conclusion:**

It can be concluded that even though the suction socket could create better suspension, fit, and gait performance, overall satisfaction was higher with the pin/lock system due to easy donning and doffing of the prosthesis.

**Trial Registration:**

irct.ir IRCT2014012816395N1

## Introduction

Suspension systems are necessary components of lower limb prostheses as they help to ensure secure coupling between the residual and prosthetic limbs [1]. Proper fit of the stump inside the prosthetic socket and appropriate selection of prosthetic suspension have positive effects on amputees’ gait, and can decrease energy consumption during ambulation [1]–[4]. Symmetry between the limbs represents a healthy gait and is one of the primary objectives of rehabilitation for lower limb amputees [5]. The gait pattern of a person with lower limb amputation is not as symmetrical as that of healthy individuals in terms of ground reaction force (GRF), time, distance of walking and joint angles [4], [6]. Among these parameters, the GRF is defined as the percentage of body weight applied to the limb during the stance phase of gait and the force that is generated for forward propulsion [7]. Bateni et al. (2002) reported that there was a higher range of motion in the hip and knee on the prosthetic side than the sound limb in transtibial amputees during walking. Moreover, the step length was longer than the sound limb due to the shorter stance time on the prosthetic side [4]. In the rehabilitation of lower limb amputees, one of the main goals is to improve the amputees’ gait pattern so that it appears as similar to gait of healthy individuals as possible. As such, many researchers have used three-dimensional motion analysis to investigate the gait parameters of transtibial amputees during different activities using various prosthetics components [4], [8], [9]. Therefore, gait analysis system might be used as a diagnostic tool to make decisions for the rehabilitation protocols.

Suspension systems play fundamental roles in prosthetic function and patient’s satisfaction [10]. Silicone liners (with total surface bearing socket (TSB)) are the most favorable form of suspension system as they provide better suspension, fit, and function during ambulation when compared with the more traditional systems, such as patellar tendon bearing (PTB) socket with Pelite liners [10]–[14].

**Figure 1:**
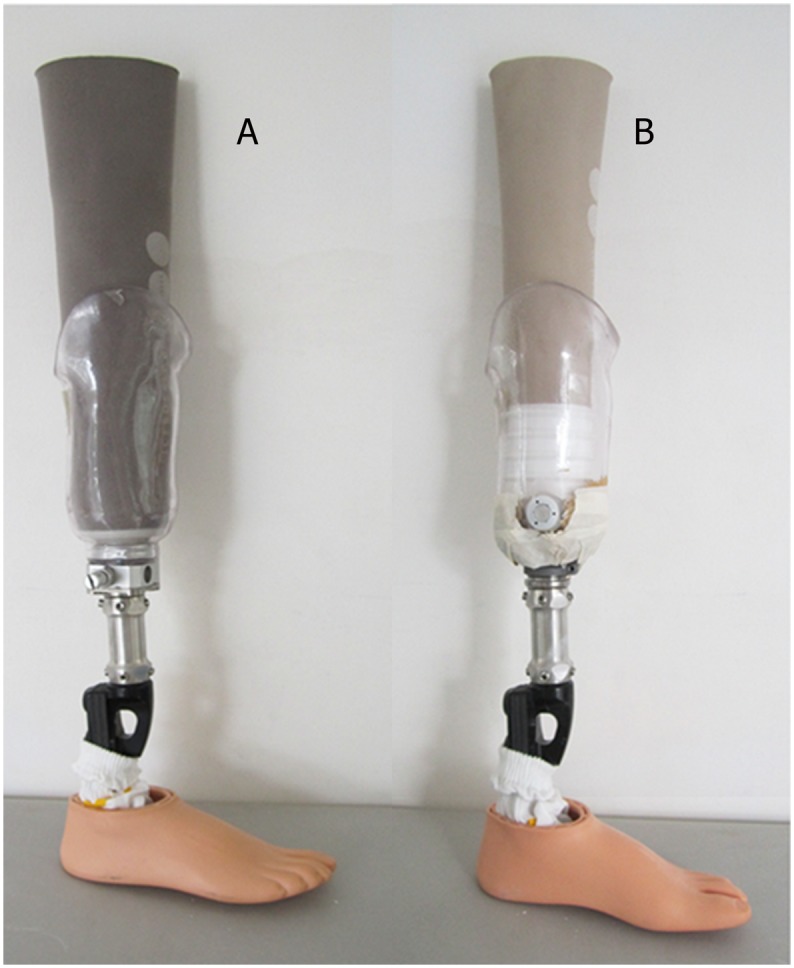
Prostheses used in this study; (A): pin/lock system (using Dermo liner) with shuttle lock, (B): suction system (using Seal-In X5 liner) with valve.

Prosthetic suspension using the Seal-in liner and valve can improve socket fit and decrease pistoning movement (vertical movement) within the socket more successfully than the pin/lock system with Dermo liner (Figure 1) [15]–[17]. Recently, Bruneli et al. (2013) reported that the Seal-in liner caused a reduction in the energy cost of walking compared with the suction socket and sleeve, especially when walking on the slope. Furthermore, the amputees could walk faster with the Seal-in suspension system, although no statistical significance was observed [14].

Amputees are generally more satisfied with the pin/Lock system due to the ease with which the liner can be donned and doffed compared with the Seal-in liner. Based on the literature, only a few studies evaluated the effects of different transtibial suspension systems on gait performance [18]–[20]. To the authors’ knowledge, no study has previously compared the effects of these two suspension systems on amputees’ gait performance.

The main purpose of this study was to compare effects of the two suspension systems on various gait parameters in unilateral transtibial amputees during level walking. The main hypothesis of the study was that the suction socket has positive influence on amputee’s gait performance, especially at the first peak GRF. Furthermore, amputees can walk more natural and symmetrical with less knee and hip flexion when using the Seal-in liner.

## Method

### Subjects

Ten unilateral transtibial amputees were found eligible to participate in this study as a sample of convenience. The subjects’ characteristics are shown in Table 1 [17]. Ethical approval was obtained from the University of Malaya Medical Centre (UMMC) Ethics Committee. All the subjects were required to sign a written consent form.

**Table 1:** Characteristics of the participants.

Subject no.	Age	Height (cm)	Mass (Kg)	Cause of amputation	Amputated side	Stump length (cm)^1^	Mobility grade^2^
1	45	168	75	Diabetic	Left	14	K2
2	35	173	90	Trauma	Left	15	K3
3	22	168	60	Trauma	Left	14	K3
4	71	181	75	Diabetic	Left	13.5	K2
5	49	167	64	Trauma	Right	13	K3
6	37	177	99	Diabetic	Right	17	K2
7	51	160	57	Diabetic	Right	14	K3
8	52	165	60	Diabetic	Left	15	K3
9	62	169	72	Trauma	Right	13	K2
10	34	172	86	Trauma	Left	16	K3

1 Stump Length: Inferior edge of patella to distal end of the stump.

2 Based on the American Academy of Orthotists & Prosthetists [21].

The inclusion criteria for the study consisted of unilateral transtibial amputation, walking without walking aids [21], steady limb volume during the previous year, pain- and ulcer-free stump, and stump length of more than 11 cm. The latter was considered optimal for use of the Seal-in transtibial liner, as stated by the manufacturer [22].

We registered our trials retrospectively (IRCT2014012816395N1) with Iranian Registry of Clinical Trials (http://www.irct.ir/searchresult.php?id=16395&number=1) as when we started this project, these two systems (seal-in and dermo liner) were already in the market and were being used by many amputees around the world. Also these two systems got CE and were produced based on ISO 9001 and ISO 13485 quality system standards. Furthermore, ethic Committee at university of Malaya did not considered the study as clinical trial. We will prospectively register other clinical trials in future.

The protocol for this trial is available as supporting information; see Protocol S1.”

### Procedures

As the participants were using different suspension systems (such as PTB or TSB) prior to the study, a single registered prosthetist designed and aligned two transtibial prostheses for each subject to prevent any bias in the results. Only the suspension systems were different while all other components including feet were similar for both prostheses. One prosthesis used the Iceross Dermo Liner with shuttle lock (pin/lock system) while the other used the Iceross Seal-in liner with valve (suction system) [15], [17]. The subjects used Flex-Foot and the two suspension systems (Seal-in and Dermo liner) for the first time in this study. This study was not blinded as our subjects easily could distinguish between the suspension systems.

Prior to the experiment, the subjects participated in gait training for the new prostheses. This took place in the Brace and Limb laboratory (Department of Biomedical Engineering, University of Malaya, Malaysia) [15], [17]. The prosthetist ensured similar lower limb height and toe-out angle, and that there was no gait deviation. Bench alignment and dynamic alignment during standing and walking was carried out. A four-week acclimation period was allocated for each prosthetic leg. The subjects used identical shoes during training and experiments.

Kinematic and kinetic gait evaluations were completed using the Vicon 612 system (7 MXF20 motion capture cameras; Plug-in-Gait, Oxford Metrics; Oxford, UK). The data collection frequency was set at 50 Hz for the synchronized cameras and two force plates (Kistler). Sixteen reflective markers were attached to the subjects’ prosthetic and sound lower limbs (according to the Helen Hayes marker set). The knee and tibia markers for the prosthetic limb were affixed to the lateral proximal and lateral distal socket walls, respectively. Following this, each subject completed five gait trials at a self-selected pace for each suspension system. A trial was considered to be appropriate provided that both feet landed properly on the force plates (whole foot was on the force plate). In order to determine proper landing on the force plate, a video recorder was used and an assistant stood one meter away from the force plate to check the foot position. All the subjects were asked to walk at their most comfortable speed in the motion laboratory on 10-meter walkway [18].

The 10-meter walkway is a common practice in research studies [18]. Prior to the test, the participants were asked to practice walking in the experiment setting in order to accustom to the environment. Proper landing of the foot on the force plate proved to be challenging (due to masking of the force plates’ location); therefore, sometimes the participants were required to repeat the trials. Nevertheless, we did not inform them which trial is proper or why they were asked to repeat a trial. To minimize the effect of fatigue, the participants were allowed to take rest whenever necessary. During the pilot study, it became apparent that when the patients became tired, the speed of gait was not consistent between the trials. The pin/lock system was tested first followed by the suction socket for all the amputees to ensure consistency.

Finally, to evaluate the effect of these two suspension systems on patients’ satisfaction, parts of the PEQ questionnaire were utilized. The PEQ questionnaire consists of 82 items grouped into nine subscales. Based on Legro et al., each question in the scales could be used separately [12]. We decided to use some questions that were more relevant to the suspension system. To compare the two systems, we assessed the subjects’ satisfaction with donning and doffing the prosthesis, walking (level surface, unlevel ground), stairs (ascending and descending), sitting, fit, cosmetic and one question to ask overall satisfaction with each system. Regarding the problems with suspension systems, the participants responded to the following questions: unwanted sounds, swelling [edema], pain, skin irritation, pistoning or movement inside the socket, smell, wound and sweat inside the socket [17].

### Data Analysis

As the walking speed was inconsistent, the data for each time frame was normalized to the whole stride time [23]. The vertical and fore-Aft GRF were also normalized to the body mass.

As symmetry is indicative of normal gait, the symmetry index (SI) was used to compare non-amputated and amputated limbs [6], [24] with the pin/lock and the suction socket [25], [26]. To calculate SI, a modified equation from the work of Herzog et al. (1989) was used:
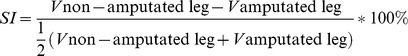



In this formula, ***V***_amputated leg_ represents data for the amputated leg during gait (for different gait parameters such as step length, swing time, etc) and ***V***_non-amputated leg_ is the data for the sound limb. The value of SI indicates how much the variables (amputated leg and non-amputated leg) are similar. The value of 0 shows that the two variables are completely similar, or the symmetry is perfect. Based on Astrom and Stenstrom, up to 10% was considered good symmetry [18]. The following variables were calculated (Table 2): step length, walking speed, stance and swing time (percentage), ground reaction force (GRF), fore-aft GRF, hip, knee and ankle range of motion during stance and swing [27].

**Table 2:** Average and standard deviation (in bracket) of gait parameters in ten transtibial amputees during level walking at a self-selected speed.

Parameters	Suction (Seal-In)		Symmetry (%)	Pin/Lock (Dermo)		Symmetry (%)
	Prosthetic Limb	Sound Limb		Prosthetic Limb	Sound Limb	
Step length (m)	0.61 (0.06)	0.57 (0.05)	−6.8	0.62 (0.05)	0.54 (0.04)	−13.8
Stride length (m)	1.2 (0.09)		0	1.1 (0.08)		0
Walking speed (m/s)	0.94 (0.05)		0	0.93 (0.06)		0
Stance time (% of gait cycle)	62.3 (2.4)	65.6 (2.5)	5.2	61.7 (1.6)	66.7 (1.6)	7.8
Swing time (% of gait cycle)	37.7 (2.3)	34.4 (2.5)	−9.2	38.3 (1.7)	33.3 (1.5)	−14.0
Hip position at initial foot contact (°)	32.8 (2.1)	35.9 (3.6)	9.0	33.2 (3.4)	32.6 (1.9)	−1.8
Maximum hip extension (°)	3.0 (1.8)	−2.1 (1.0)	−200	2.6 (1.5)	−2.4 (2.1)	−200.0
Hip range (°)	37.3 (2.8)	38.4 (3.4)	2.9	36.1 (2.8)	37.2 (3.0)	3.0
Knee position at initial foot contact (°)	5.4 (4.6)	1.4 (1.0)	−117.6	5.7 (3.6)	4.1 (2.5)	−32.7
Maximum knee flexion at stance (°)	13.7 (2.9)	15.1 (1.7)	9.7	12.5 (3.4)	13.4 (4.1)	6.9
Maximum knee flexion during swing (°)	75.4 (2.4)	55.1 (3.1)	−31.1	66.9 (3.9)	52.5 (3.7)	−24.1
Knee range of motion (°)	70.7 (3.5)	56.1 (2.2)	−23.0	61.5 (3.2)	52.6 (3.1)	−15.7
Ankle position at initial foot contact (°)	−0.8 (1.5)	2.1 (1.0)	200.0	0.2 (1.1)	−4.2 (1.3)	−200.0
Maximum ankle plantar flexion at stance (°)	−7.2 (2.4)	−6.6 (3.1)	−8.7	−5.9 (3.4)	−5.9 (2.7)	0.0
Maximum ankle dorsiflexion at stance (°)	14.5 (2.3)	7.3 (1.9)	−66.1	15.1 (1.3)	8.1 (2.4)	−60.3
Maximum ankle plantar flexion at swing (°)	0.3 (0.6)	−13.2 (2.9)	200.0	1.4 (1.8)	−12.1 (0.9)	200.0
Ankle range of motion (°)	21.7 (2.2)	20.7 (3.6)	−4.7	20.9 (3.2)	20.1 (1.9)	−3.9
Vertical GRF, 1^st^ peak (N)	99.7 (3.8)	121.1(2.4)	19.4	104.2 (4.2)	121.7 (2.7)	15.5
Vertical GRF, 2^nd^ peak (N)	102.6 (4.9)	101.9 (3.1)	−0.7	101.1 (3.9)	99.0 (2.4)	−2.0
Fore-aft GRF, 1^st^ peak (N)	5.4 (1.0)	7.8 (1.8)	36.4	4.6 (2.8)	9.3 (2.1)	67.6
Fore-aft GRF, 2^nd^ peak (N)	−8.0 (1.7)	−7.5 (1.5)	−6.5	−8.1 (1.1)	−7.1 (1.4)	−13.2

Statistical data was analyzed using SPSS 17.0 and *p*-values of 0.05 or less reflected statistical significance. Paired-samples t-test was employed to compare the effect of two systems on gait variables. The statistical tests were applied to all gait variables independently for both suspension systems as well as amputees’ sound limb. Moreover, the average of obtained data for each gait parameter through five successful trials was calculated for both suspension systems. Lastly, the overall average of gait parameters was calculated for all the participants to compare the suspension systems.

## Results

The mean age, height, and weight of the participants were 45.8 (SD, 14.4) years, 170 (SD, 6) cm, and 73.8 (SD, 14.2) kg, respectively. The mean stump length was 14.5 (SD, 1.3) cm and the causes for amputation were trauma and diabetes (Table 1).

The study results showed that step length and swing time on the prosthetic side were longer than that of the sound limb with both suspension systems, and that the prosthetic and sound limbs behaved significantly different (*p*<0.03) (Table 2). In addition, stance time was shorter for the prosthetic limb than the sound limb.

Maximum knee flexion during the swing phase was 75.4° and 66.9° for the suction and pin/lock systems, respectively. Also, there was a significant difference between the two systems (*p*<0.04). There was asymmetry in ankle dorsiflexion and plantar flexion at stance and swing phase between the sound and prosthetic limbs.

Significant differences (*p*<0.03) were identified in the vertical ground reaction force between the two systems only at the first peak (loading response). Asymmetry in timings of the first peak was observed with the pin/lock system. Weight transfer during the transition from double to single limb support occurred in a shorter period for the sound limb in comparison to the prosthetic limb. Furthermore, data analysis showed significantly higher magnitude of the first peak vertical GRF between the sound limb and prosthetic side with both suspension systems (*p*<0.000).

Satisfaction surveys revealed that transtibial amputees are more satisfied with the pin/lock system. Donning and doffing the prosthesis with the pin/lock system was also easier compared with the Seal-in or suction system. Nevertheless, the prosthesis with the Seal-in liner was more fit with less movement inside the socket (between the liner and socket) (Table 3, Table 4).

**Table 3:** Comparison of satisfaction with the pin/lock and suction (Seal-In liner) systems.

	Fit		Donning and doffing		Sitting		Walking (level ground)		Walking (Uneven ground)		Walking (Stairs)		Cosmetic		Overall satisfaction with prosthesis	
	Mean^1^	*p* value	Mean	*p* value	Mean	*p* value	Mean	*p* value	Mean	*p* value	Mean	*p* value	Mean	*p* value	Mean	*p* value
Pin/lock	76	.003*	88	.000*	76	.447	78	.001*	75	.040*	77	.090	83	.460	86	.004*
suction	87		35		78		86		80		79		81		75	

1 Greater mean indicates higher satisfaction.

*Indicates statistically significant values.

**Table 4:** Comparison of perceived problem with the pin/lock and suction (Seal-In liner) systems.

	Sweat		Wound		Irritation		Pistoning inside the socket		Pain		Swelling (edema)		Sound		Smell	
	Mean^1^	*p* value	Mean	*p* value	Mean	*p* value	Mean	*p* value	Mean	*p* value	Mean	*p* value	Mean	*p* value	Mean	*p* value
Pin/lock	29	.080	0	-	0	-	27	.000*	29	.000*	0	-	25	.000*	4	.153
suction	27		0		0		4		16		0		4		6	

1 Greater mean indicates more complaints/problems.

*Indicates statistically significant values.

Subjective feedback showed that the participants spent more time and effort to don and doff the prosthesis with the suction socket but they did not feel any traction at the end of the residual limb, and the prosthesis acted like a natural part of their body.

**Figure 2:**
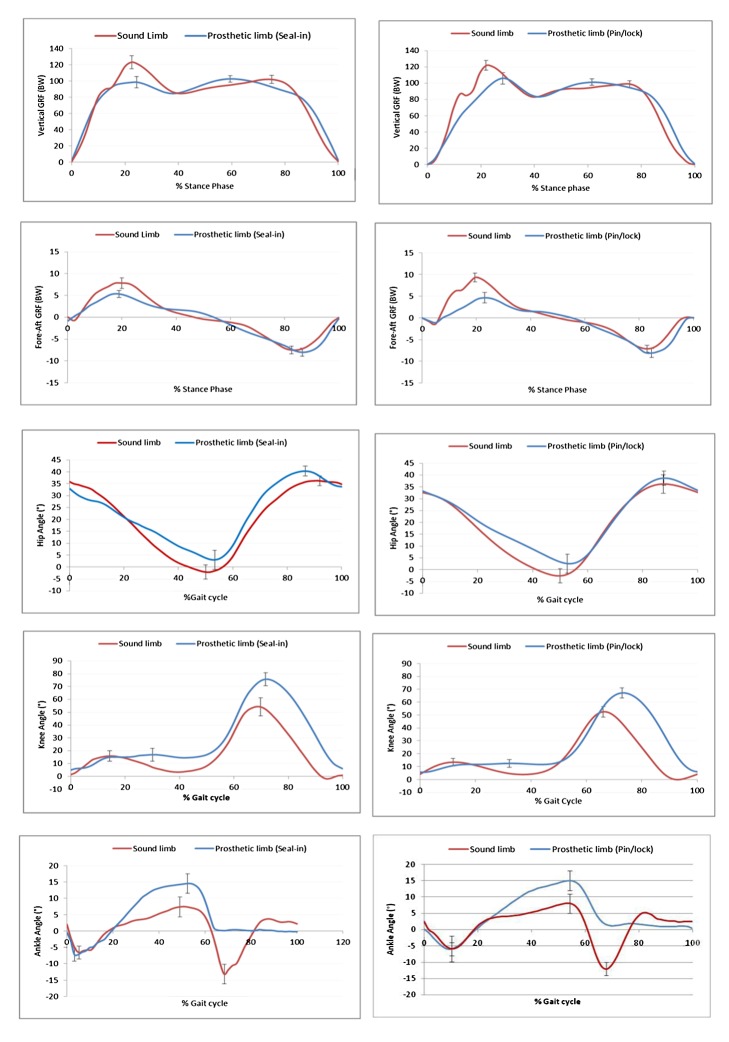
Kinematic patterns for prosthetic and intact legs with the suction (Seal-In) and pin/Lock (Dermo) suspension systems for ten participants (mean values).

**Figure 3:**
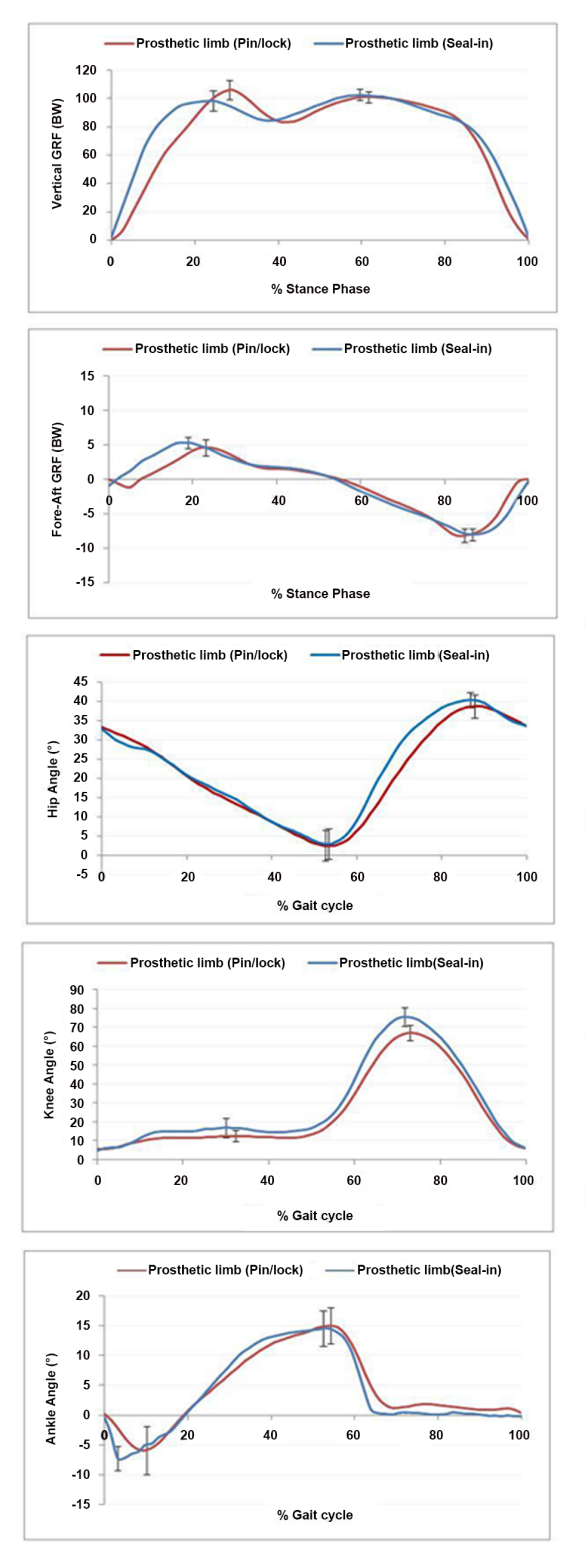
Comparison between the suction and pin/lock systems for prosthetic limb.

Table 2, Figures 2 and 3 show the average values of gait parameters and symmetry for both the suction (Seal-in) and pin/lock (Dermo) suspension systems for the ten participants.

## Discussion

In this study, two different suspension systems, the pin/lock and suction, were compared in terms of their effect on kinetic and kinematic gait parameters. The systems had been previously studied both statically and dynamically to investigate socket fit and level of pistoning within the socket [15]–[17]. The previous findings revealed that the suction suspension created a better socket fit.

Pitkin (1997) and Astorm (2004) stated that the better the socket fit is, the lower would be the asymmetry between the sound and prosthetic legs, which will result in close to normal gait in amputees [18], [28]. This study hypothesized that suction suspension can improve amputee’s gait. It was also conjectured that gait symmetry would increase with the use of suction suspension system.

### Ground Reaction Forces

Ground reaction force mirrors the external forces applied to the legs [29], [30]. Two peaks can be detected in GRF; the first peak reflects the quality of shock absorption by the locomotor system during gait. Significant differences (*p<*0.00) were found in the vertical GRF (first peak) with both suspension systems. Research findings have shown significantly higher magnitude of the first peak vertical GRF for the sound limb. Therefore, it can be deduced that the sound limb can bear more load than the prosthetic limb during loading response [4], [31]. The magnitude of first peak for sound limb in both systems was similar to the average magnitude in normal people [32], [33].

Figure 2 shows an asymmetry in timings of the first peak with the pin/lock system for the sound limb compared with the amputated leg when using suction or the Seal-in suspension system. This may indicate that the weight shift happened over shorter period for the contralateral limb from double to single limb support. As such, it can be implied that the participants had less confidence to bear weight (from heel strike to loading response) on the prosthetic side when using the pin/lock system. This finding also provides good evidence to support the previous questionnaire surveys [14]–[16], [34] that revealed more confidence when using the prosthetic device with the suction socket.

Moreover, vertical GRF graphs revealed that the midstance time on the prosthetic side (using suction or pin/lock system) was shorter than the sound side. Also, there was no significant difference in the magnitude of second GRF between the sound and prosthetic legs for none of the suspension systems. It might be interpreted that the subjects could bear similar loads on both the sound and prosthetic legs (with both systems) from midstance to toe off.

By looking at the pattern of resultant fore-aft GRF, similar acceleration forces (horizontal propulsive force) are evident for both legs; nevertheless, deceleration force (braking force toward posterior) is larger at the sound limb. Previous findings confirm this observation with some slight differences in magnitudes that can be attributed to the variations in walking speed, prosthetic components and prosthetic foot. Not only the magnitude of deceleration force, but also the duration was dissimilar between the legs, with the sound limb having a shorter duration than the prosthetic side. Zmitrewicz et al. (2006) reported similar findings [35]. It is also worth mentioning that the deceleration force appeared later in the gait cycle for the prosthetic limb, especially with the pin/lock system. As it was hypothesized, this may suggest that the participants were more confident to bear weight on the sound limb.

The propulsion forces with both the suction and pin/lock systems were of similar magnitudes for both the sound and prosthetic limbs. Propulsive forces contribute to steady speed of walking, balanced loading and symmetrical gait pattern. The observed constant magnitudes of propulsion forces for the sound and prosthetic limbs signified a good balance (symmetry) between the legs, particularly with the suction suspension system.

### Temporal-spatial Parameters

Time-distance parameters provide information about position and timing of gait. The temporal-spatial results with the two suspension systems supported the findings of previous research [5], [32], [33]. Prosthetic gait is distinguished by longer step length, lower walking speed, higher cadence and higher swing time when compared with both normal individuals and the amputee’s sound leg [32], [33], [36].

In the current study, both suspension systems caused longer step length on the prosthetic side. Therefore, it can be interpreted as longer period of swing phase, which would be accompanied by longer time of load bearing on the contralateral limb. Amputees adopt longer step lengths on prosthetic limbs to off-load the amputated side. There was also significant differences between the prosthetic and sound limbs with the suction (*p*<0.05) and pin/lock systems (*p*<0.02).

Walking speed indicates the ability to transfer load from one leg to another, and to preserve forward momentum of body mass. This study revealed that subjects walked at a speed of 0.94 m/s and 0.93 m/s when using the suction and pin/lock systems, respectively. TTB amputees walk at lower speed compared with able-bodied individuals (1.2–1.5 m/s) [5], [32], [33], [37]. The tendency to walk at slightly higher speed when using the suction system is possibly due to the fact that the subjects had more confidence with the prosthesis.

### Joint Angle

Information on the angular and linear motions of the body segments is gained from the kinetic data. Both prosthetic (pin/lock and suction) and sound limbs were found to have similar angular motion at the hip and knee. The most remarkable difference was observed at the ankle joint. This finding is in line with previous studies on different prosthetic ankle types, which also determined that the ankle affected the degree of control over the prosthesis [38]–[40]. Gait progression is altered when the anatomical ankle joint is missing, as the ankle plantar flexion generates over 80% of the mechanical power during normal walking. Not all prosthetic foot designs can compensate this action; therefore, various prosthetic feet result in different ankle joint angles.

The knee range of motion with the pin/lock system was more consistent between the prosthetic and sound limbs than with the suction socket (61.5, 52.5 vs. 70.7, and 56.1, respectively), and there was significant difference between the two systems.

There was asymmetry between ankle angles for right and left legs using both systems, especially at the end of the stance and preswing phases. Maximum dosiflexion at the stance phase reached 14.5 and 15.1 degrees in the suction and pin/lock systems, respectively. This was possibly due to more flexibility in the prosthetic foot.

During training in the prosthetic laboratory, all the subjects stated that the Talux foot was more comfortable than the foot they usually used, especially during heel strike and push off. They claimed that the foot acted like a spring, and that it helped them to walk faster [17].

### PEQ

Satisfaction with prosthestic device could be influenced by several factors. A research study by Legro et al. on 92 amputees over 27 months revealed that prosthetic fitting influenced amputees’ satisfaction with their device [12]. Moreover, easy donning and doffing might have a positive effect on subjects’ satisfaction with prosthetic device [12], [17]. The suction system could increase socket fit and resolve the so-called problem of “milking” (distal tissue stretch caused by the pin and lock). This milking phenomenon can also result in pain, particularly at the distal end of the residual limb. Other factors such as easy donning and doffing could also influence amputees’ satisfaction [17]. The findings of the current study showed that despite the higher observed fitting, the participants preferred to use the pin/lock system in long term as it was easier to don and doff than the suction system.

### Study Limitations

Many factors can affect amputees’ gait and satisfaction. This study was conducted on a small sample size and this may have impact on the statistical relevance of the results. Additionally, more suspension alternatives should be studied in future to deepen insights into the effectiveness and comfort of suspension systems. It is also worth investigating effects of the available suspension systems on proprioception.

## Conclusions

From the outcome of this study, it can be concluded that amputee’s gait performance was positively influenced by the Seal-in liner due to better suspension and fit within the socket. Nevertheless, overall satisfaction with prosthesis was higher with the pin/lock system due to easy donning and doffing. Good prosthetic suspension system must secure the residual limb inside the prosthetic socket and make donning and doffing procedures easier. Further research is needed to evaluate more amputees, and to offer a guideline for proper selection of suspension system.

## Supporting Information

Protocol S1 (DOC)

## References

[1] Baars E, Geertzen J (2005) Literature review of the possible advantages of silicone liner socket use in trans-tibial prostheses. Prosthet Orthot Int. 29: 27–37. 10.1080/17461550500069612 16180375

[2] Ku PX, Abu Osman NA, Yusof A, Wan Abas WAB (2012) The Effect on Human Balance of Standing with Toe- Extension. PLoS ONE. 7(7): 1–5. 10.1371/journal.pone.0041539 PMC3405114 22848523

[3] Czerniecki JM, Gitter AJ (1996) Gait analysis in the amputee: Has it helped the amputee or contributed to the development of improved prosthetic components? Gait Posture. 4(3): 258–268.

[4] Bateni H, Olney S (2002) Kinematic and kinetic variations of below knee amputee gait. J Prosthet Orthot. 14: 2–10.

[5] Isakov E, Keren O, Benjuya N (2000) Trans-tibial amputee gait: time–distance parameters and EMG activity. Prosthet Orthot Int. 24: 216–20. 10.1080/03093640008726550 11195356

[6] Robinson RO, Herzog w, Nigg BM (1987) Use of force platform variables to quantify the effect of chiropractic manipulation on gait symmetry. J Manipulative Physiol Ther. 10(4): 172–176. 2958572

[7] Kishner S (2010) Gait analysis after amputation. Medscape http://emedicine.medscape.com/article/1237638-overview (accessed 2013 February 12).

[8] Sanderson D, Martin PE (1997) Lower extremity kinematic and kinetic adaptations in unilateral below knee amputees during walking. Gait Posture. 126–136.

[9] Rusaw D, Ramstrand N (2011) Motion-analysis studies of transtibial prosthesis users: a systematic review. Prosthet Orthot Int. 35: 8–19. 10.1177/0309364610393060 21515885

[10] Eshraghi A, Abu Osman NA, Gholizadeh H, Karimi MT, Ali S (2012) Pistoning Assessment in Lower Limb Prosthetic Sockets. Prosthet Orthot Int. 36: 15–24. 10.1177/0309364611431625 22269941

[11] Baars E, Geertzen J (2005) Literature review of the possible advantages of silicon liner socket use in trans-tibial prostheses. Prosthet Orthot Int. 29(1): 27–37. 10.1080/17461550500069612 16180375

[12] Legro MW, Reiber G, Aguila MD, Ajax MJ, Boone DA, et al (1999) Issues of importance reported by persons with lower limb amputations and prostheses. J Rehabil Res Dev. 36(3): 155–63. 10659798

[13] Heim M, Wershavski M, Zwas ST, Siev-Ner I, Nadvorna H, et al (1997) Silicone suspension of external prostheses. A new era in artificial limb usage. J Bone Joint Surg Br. 79(4): 638–640. 10.1302/0301-620x.79b4.7350 9250755

[14] Brunelli S, Delussu AS, Paradisi F, Pellegrini R, Traballesi MA (2013) comparison between the suction suspension system and the hypobaric Iceross Seal-In X5 in transtibial amputees. Prosthet Orthot Int. 37(6), 436–444. 10.1177/0309364613476531 23436696

[15] Gholizadeh H, Abu Osman NA, Kamyab M, Eshraghi A, Wan Abas WAB, et al (2012) Transtibial prosthetic socket pistoning: Static evaluation of Seal-In X5 and Dermo Liner using motion analysis system. Clin Biomech. 27: 34–39. 10.1016/j.clinbiomech.2011.07.004 21794965

[16] Gholizadeh H, Abu Osman NA, Kamyab M, Eshraghi A, Lúðvíksdóttir ÁG, et al.. (2012) Clinical evaluation of two prosthetic suspension systems in a bilateral transtibial amputee. Am J Phys Med Rehabil. 91(10), 894–898. 10.1097/PHM.0b013e31823c74d7 22173083

[17] Gholizadeh H, Abu Osman NA, Eshraghi A, Ali S, Sævarsson SK, et al (2012) Transtibial prosthetic suspension: less pistoning versus easy donning and doffing. J Rehabil Res Dev. 49(9): 1321–30. 10.1682/jrrd.2011.11.0221 23408214

[18] Astrom I, Stenstrom A (2004) Effect on gait and socket comfort in unilateral trans-tibial amputees after exchange to a polyurethane concept. Prosthet Orthot Int. 28: 28–36. 10.3109/03093640409167922 15171575

[19] Board WJ, Street GM, Caspers CA (2001) comparison of transtibial amputee suction and vacuum socket conditions. Prosthet Orthot Int. 25(3): 202–209. 10.1080/03093640108726603 11860094

[20] Boutwell E, Stine R, Hansen A, Tucker K, Gard S (2012) Effect of prosthetic gel liner thickness on gait biomechanics and pressure distribution within the transtibial socket. J Rehabil Res Dev. 49: 227–240. 10.1682/jrrd.2010.06.0121 22773525

[21] American Academy of Orthotists & Prosthetists. Medicare. PSC044: Medicare guideline forms: K-level determination; 2010. http://www.oandp.org/bookstore/products/PSC044.asp Accessed November 8, 2011

[22] Össur (2008) Compatibility and the perfect fit. Isn’t this how all great relationships start? Available at: http://www.ossur.com/lisalib/getfile.aspx?itemid=17635. Accessed 2011 May 20.

[23] Farahmand F, Rezaeian T, Narimani R, Dinan PH (2006) Kinematic and dynamic analysis of the gait cycle of above-knee amputees. Scientia Iranica, 13 (3): 261–271.

[24] Herzog W, Nigg BM, Read LJ, Olsson E (1989) Asymmetries in ground reaction force patterns in normal human gait. Med Sci Sports Exerc. 21, 110–114. 10.1249/00005768-198902000-00020 2927295

[25] Baker PA, Hewison SR (1990) Gait Recovery Pattern of Unilateral Lower Limb Amputees During Rehabilitation. Prosthet Orth Int. 14 (2): 80–84. 10.3109/03093649009080327 2235305

[26] Chow DHK, Holmes AD, Lee CKL, Sin SW (2006) The effect of prosthesis alignment on the symmetry of gait in subjects with unilateral transtibial amputation. Prosthet Orth Int. 30(2), 114–128. 10.1080/03093640600568617 16990222

[27] Winter DA, Sienko SE (1988) Biomechanics of below knee amputees. J Biomech. 21, 361–367. 10.1016/0021-9290(88)90142-x 3417688

[28] Pitkin MR (1997) Model of residuum-socket interface. Proceedings, 23rd Annual Meeting & Scientific Symposium, American Academy of Orthotists and Prosthetists, 1997, San Francisco, 21–2.

[29] Engsberg JR, Lee AG, Tedford KG, Harder JA (1993) Normative ground reaction force data for able-bodied and trans-tibial amputee children during running. Prosthet Orth Int. 17, 83–89. 10.3109/03093649309164361 8233773

[30] Stergiou N, Giakas G, Byrne JE, Pomeroy V (2002) Frequency domain characteristics of ground reaction forces during walking of young and elderly females. Clin Biomech. 17, 615–617. 10.1016/s0268-0033(02)00072-4 12243722

[31] Vanicek N, Strike S, McNaughton L, Polman R (2009) Gait patterns in transtibial amputee fallers vs. non-fallers: Biomechanical differences during level walking. Gait Posture. 29, 415–420. 10.1016/j.gaitpost.2008.10.062 19071021

[32] Winter DA (1991) Kinematic and kinetic patterns in human gait: normal, elderly and pathological. University of waterloo press, Ontario, Canada.

[33] Perry J, Davids JR (1992) Gait analysis: normal and pathological function. J Pediatr Orthoped, 12(6), 815.

[34] Ali S, Abu Osman NA, Morteza N, Eshraghi A, Gholizadeh H, et al.. (2012) Clinical investigation of the interface pressure in the trans-tibial socket with Dermo and Seal-In X5 liner during walking and their effect on patient satisfaction. Clin Biomech. 27(9), 943–948 10.1016/j.clinbiomech.2012.06.004 22795863

[35] Zmitrewicz RJ, Neptune RR, Walden JG, Rogers WE, Bosker GW (2006) The effect of foot and ankle prosthetic components on braking and propulsive impulses during transtibial amputee gait. Arch Phys Med Rehabi. 87, 1334–1339. 10.1016/j.apmr.2006.06.013 17023242

[36] Nolan L, Wit A, Dudziñski K, Lees A, Lake M, et al.. (2003) Adjustments in gait symmetry with walking speed in trans-femoral and trans-tibial amputees. Gait Posture. 17, 142–151. 10.1016/s0966-6362(02)00066-8 12633775

[37] Moosabhoy MA, Gard SA (2006) Methodology for determining the sensitivity of swing leg toe clearance and leg length to swing leg joint angles during gait. Gait Posture. 24(4): 493–501. 10.1016/j.gaitpost.2005.12.004 16439130

[38] Marinakis GNS (2004) Inter limb symmetry of traumatic unilateral transtibial amputees wearing two different prosthetic feet in the early rehabilitation stage. J Rehabil Res Dev. 41, 581–590. 10.1682/jrrd.2003.04.0049 15558386

[39] Vanicek N, Strike S, McNaughton L, Polman R (2009) Gait patterns in transtibial amputee fallers vs. non-fallers: Biomechanical differences during level walking. Gait Posture. 29, 415–420. 10.1016/j.gaitpost.2008.10.062 19071021

[40] Collins SH, Kuo AD (2010) Recycling Energy to Restore Impaired Ankle Function during Human Walking. PLoS ONE. 5(2): e9307. 10.1371/journal.pone.0009307 PMC2822861 20174659

