# Supplementary material for: The Effects of Suction and Pin/Lock Suspension Systems on Transtibial Amputees’ Gait Performance
Source: PLoS One. 2014 May 14;9(5):e94520. doi: 10.1371/journal.pone.0094520 (PMC4021017; doi:10.1371/journal.pone.0094520)
Supplement: Protocol S1 — (DOC) [file pone.0094520.s001.doc]

**Protocol S1**

**Title of the study**: The Effects of Suction and Pin/Lock Suspension Systems on Transtibial Amputees’ Gait Performance

This study aimed to obtain kinematics and kinetics of trans-tibial amputees gait using the Seal-In Liner and Dermo Liner (both are considered silicone liners). Ethical approval was obtained from the University of Malaya Medical Centre Ethics Committee. All the subjects were required to sign a written consent form. The inclusion criteria for the study consisted of unilateral transtibial amputation, walking without walking aids, steady limb volume during the previous year, pain- and ulcer-free stump, and stump length of more than 11 cm. The latter was considered optimal for the use of the Seal-In transtibial liner as stated by the manufacture. A single registered prosthetist designed and aligned two transtibial prostheses for each subject to prevent any bias in the results. Only the suspension systems were different and all other components including the prosthetic feet were alike for both prostheses. A four-week acclimation period was allocated for each prosthetic leg. Following this, each subject completed five gait trials at a self-selected pace for each suspension system**.**

**Study Protocols**

1. Consent forms were given to the subject. After ensuring that the consent form was signed, the subject information sheet was provided for the subject and potential question(s) were answered.
2. This was the clinical trial phase of the overall study:

i.) Study population: 10 subjects of trans- tibial amputees using prosthesis. (Volunteer from Department of Rehabilitation Medicine)

ii.) Inclusion criteria

Subject was a trans-tibial (male or female) with a normal stump, without any contracture, or diabetes

Subject was currently using prosthesis for more than 1 month.

Subject was able to give consent for the study

3. Two different prosthesis were fabricated for each subject with each of the two suspension systems.

4. Subjects were required to walk with two different prosthetic suspension systems in the Brace and Limb Laboratory (Clinical P&O Lab.) and do various physical activities, e.g. donning and doffing of prosthesis, stand up, sit down, standing, sitting, stair and ramp climbing.

5. The researcher (Certified Prosthetist and Orthotist) checked the prosthesis alignment and fitness of the prosthesis socket.

6. Subjects were asked to wear each of the two prostheses for one month and come back after one month for evaluation of their gait and in-socket pressure in the Motion Analysis Laboratory, and for the PEQ questionnaire survey.

The collected data was analyzed and presented in a form that reflected the participants’ daily activities for the past one month. All data was kept confidential and only used for research purpose. Ownership of the prosthesis at any time will never be affected by this study.

**Contact person:** Hossein Gholizadeh, Dept of Biomedical Engineering, Faculty of Engineering, University of Malaya (Email: gholizadeh@um.edu.my)
